# Metal Surface Modification for Obtaining Nano- and Sub-Nanostructured Protective Layers

**DOI:** 10.1186/s11671-017-1964-5

**Published:** 2017-03-09

**Authors:** Volodymyr Ledovskykh, Yuliya Vyshnevska, Igor Brazhnyk, Sergiy Levchenko

**Affiliations:** 1 National Aviation University, 1, Kosmonavta Komarova Ave, Kyiv, 03058 Ukraine; 2 National Technical University of Ukraine “Igor Sikorsky Kyiv Polytechnic Institute”, 37K20, Peremohy Ave, Kyiv, 03056 Ukraine; 3 Institute for Renewable Energy, 20A, H. Khotkevych Str., Kyiv, 02094 Ukraine; 4 Gimasi SA Ukraine R&D Centre, 18, Via Luigi Lavizzari, Mendrisio, 6850 Switzerland

**Keywords:** Metal protection, Surface engineering, Pitting corrosion, Isomolar series, Complexes, Phase layers

## Abstract

Regularities of the phase protective layer formation in multicomponent systems involving inhibitors with different mechanism of protective action have been investigated. It was shown that optimization of the composition of the inhibition mixture allows to obtain higher protective efficiency owing to improved microstructure of the phase layer. It was found that mechanism of the film formation in the presence of NaNO_2_-PHMG is due to deposition of slightly soluble PHMG-Fe complexes on the metal surface. On the basis of the proposed mechanism, the advanced surface engineering methods for obtaining nanoscaled and sub-nanostructured functional coatings may be developed.

## Background

Protection of metals under different conditions in various environments remains the important issue for science and technology. Recent advances in research of the nanoscaled systems allow the development of simple and inexpensive methods for obtaining nanostructured protective coatings with improved functional properties [1–5].

The protection mechanism of certain types of corrosion inhibitors is based on the formation of the phase protective layers on the metal surface. Among them, one may list the inorganic passivators that facilitate the formation of the dense oxide or salt protective films and complexing type inhibitors that are forming phase metalorganic layers. The mechanism of protective activity of the complexing type inhibitors consists in the formation of slightly soluble complexes of organic ligands with the metal ions. Deposition of such compounds on the metal surface in form of the phase layers leads to the gradual decrease in corrosion rate thus providing the efficient anticorrosive protection. The crucial parameters for ensuring high protective efficiency of the complexing type inhibitors are the high stability and low solubility of complexes with the cations of the metal being protected [6, 7].

Establishing regularities of the phase layer formation processes in multicomponent systems on a nano- and sub-nanoscale will enable the development of novel surface engineering techniques for obtaining functional coatings with desired functional properties.

The present study aimed to investigate the joint action of the inhibitors with different protective mechanism as well as their influence on the phase layer microstructure and achieved anticorrosive efficiency.

## Methods

The studied materials were as follows: steels 08kp and 20, corrosion environment—aqueous saline solutions with compositions of 0.3 g/l NaCl, 0.3 g/l Na_2_SO_4_, and 0.3 g/l NaHCO_3_, inhibitors—NaNO_2_, Na_2_SiO_3_, polyhexamethylene guanidine (PHMG), and their isomolar mixtures (with total concentration of 30 mmol/l).

Corrosion tests and determination of the inhibitor efficiency were performed gravimetrically according to the standard procedure. For this purpose, specimens were exposed into corrosion environment for different times from 72 to 168 h. The ratio of solution volume (ml) to the area of metal sample (cm^2^) was a 10:1. The inhibition efficiency was determined by the equation $Z=\left[\left({K}_m-{K}_m^{\hbox{'}}\right)/{K}_m\right]\cdot 100\%$, the inhibition coefficient − by the equation $\gamma ={K}_m/{K}_m^{\hbox{'}}$, where ${K}_m,{K}_m^{\hbox{'}}-$ the corrosion rate of metal in solutions without and with inhibitor correspondingly (*K*_*m*_ = *Δm*/(*S* ⋅ *τ*), where *Δm* − the loss of the sample weight, g; *S* − the sample area, m^2^; *τ* − exposure time, h).

Polarization measurements were carried out in potentiostatic regime in a three-electrode cell with separated cathodic and anodic compartments. Carbon steel was used as the working electrode, platinum—as the counter-electrode and an Ag|AgCl|KCl (sat.) electrode—as the reference one. In this paper, the potential values are given with respect to the normal hydrogen electrode potential.

The morphological characteristics and elemental composition of the films were carried out with the scanning electron microscope (EVO-50, Zeiss, Germany) equipped with the energy-dispersive detector (INCA PENTA FET × 3, Oxford Instruments, Co., UK). Elemental analysis of the protective thin films has been carried out using Auger microprobe JAMP-9500F in the scanning electron microscopy mode.

Investigation of the complex formation process and geometry optimization has been carried out with a hybrid QM/MM approach using NWchem 6.5 computational chemistry package [8].

## Results and Discussion

**Fig. 1:**
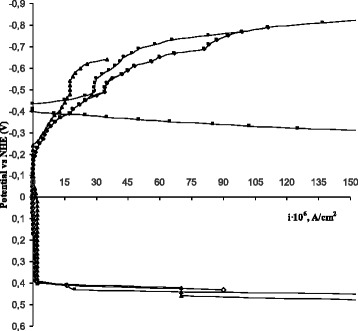
Cathodic and anodic polarization curves for carbon steel: *black square*—in background aqueous saline solution, *black up-pointing triangle*—in the presence of Na_2_SiO_3_ (30 mmol/l), *lozenge*—in the presence of NaNO_2_ (30 mmol/l), *black circle*—in the presence of synergetic mixture of NaNO_2_ (10 mmol/l) and Na_2_SiO_3_ (20 mmol/l)

The results of gravimetric tests show high protective efficiency of NaNO_2_ in aqueous saline solutions. The inhibition efficiency reaches a value of 99.2% at the sodium nitrite concentration of 30 mmol/l. The polarization measurements in the presence of 30 mmol/l NaNO_2_ show the potential shift toward the positive side that leads to increased corrosion resistance of the carbon steel. The passive zone extends on a wide range of potentials with significant drop in current density (Fig. 1).

**Fig. 2:**
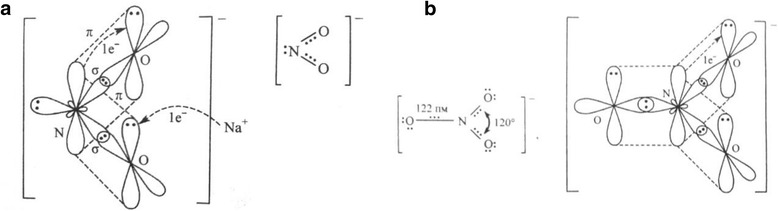
Electronic structure of the nitrite (**a**) and nitrate (**b**) ions

The protection mechanism of NaNO_2_ usually attributed to its ability to facilitate the formation of dense Fe_3_O_4_ or mixed oxide films that prevent further corrosion damage [9]. Such properties may be associated with the electronic structure of NO_2_^−^ ion due to the presence of lone pair of electrons in sp^2^-hybrid orbital that provides with the electron-donor properties in contrast to NO_3_^−^ ions that do not exhibit protective properties (Fig. 2).

**Fig. 3:**
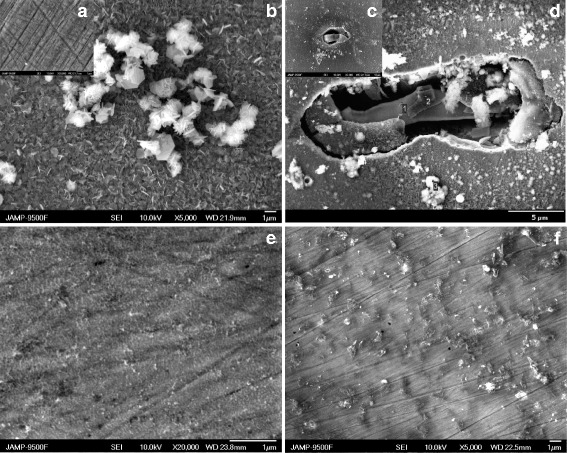
SEM images of carbon steel after exposition in **a** without exposure, **b** background aqueous saline solution, **c**, **d** presence of NaNO_2_ (30 mmol/l), **e** presence of Na_2_SiO_3_ (30 mmol/l), **f** presence NaNO_2_ (10 mmol/l) + Na_2_SiO_3_ (20 mmol/l). Exposure time, 168 h

SEM images of the samples immersed into the NaNO_2_ solution reveal the significant changes in the morphology attributed to the formation of protective film as well as signs of corrosion attack (Fig. 3 c, d). While the gravimetric measurements have shown high inhibitive efficiency, the surface morphology has significant amount of localized corrosion damage. The Auger spectroscopy has been employed to investigate the elemental composition of the protective layer. Obtained results indicate presence of the iron oxide and are found to be in a good agreement with theoretical expectations. Local types of corrosion such as pitting are considered as the most dangerous ones; hence, this drawback must be addressed during development of the inhibitive compositions based on sodium nitrite.

**Fig. 4:**
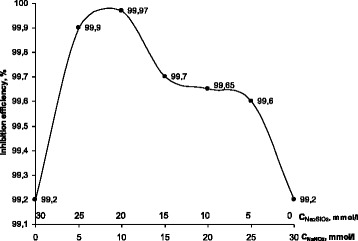
Dependence of the inhibition efficiency for carbon steel in the aqueous saline solution on the ratio of molar concentrations of the individual components in isomolar mixtures NaNO_2_-Na_2_SiO_3_ (the total concentration of the components—30 mmol/l)

To achieve the synergistic effect, the inhibitive composition should combine the components with different mechanism of the anticorrosive action [10–13]. The inhibition mechanism of Na_2_SiO_3_ is attributed to a formation of slightly soluble silicates of iron on the metal surface. The distribution of the protective efficiency within the NaNO_2_-Na_2_SiO_3_ inhibitive mixture in relation to the concentration ratio of the components has been obtained using the isomolar series method (Fig. 4). The regularity shows extreme character reaching peak value of *z* = 99.97% at the concentration ratio NaNO_2_-Na_2_SiO_3_ as 1:2, which is higher than protective efficiency of both individual components.

Polarization measurements for this mixture are found to be in a good agreement with the gravimetric studies (Fig. 1). The inhibitive composition of NaNO_2_-Na_2_SiO_3_ ensures significant drop in the current density in a wide range of potentials that correspond to a passive state of the surface. SEM images reveal significantly improved morphology of the protective film formed in presence of the NaNO_2_-Na_2_SiO_3_ mixture compared to sole sodium nitrite solution (Fig. 4f). Obtained protective film has continuous structure without signs of pitting corrosion. Elemental composition of the protective layer showed presence of the silicon that indicates direct participation of SiO_3_^2−^ ions in the film formation process. While the NaNO_2_-Na_2_SiO_3_ mixture showed significant improvement in inhibition coefficient over sole sodium nitrite, the most valuable outcome of the composition optimization is greatly enhanced efficiency toward local corrosion damage.

To ensure reliable corrosion protection for diverse applications, the inhibitive composition should provide efficient reduction in corrosion rate (in wide range of pH and) under elevated temperature conditions. At the same time, it was found that NaNO_2_-Na_2_SiO_3_ mixture demonstrates high protective efficiency at room temperature, while the protection coefficient declines as the temperature rises.

Further improvement in protective performance may be achieved by combining inorganic passivator with organic ligands that could form complexes with the metal ions. The mechanism of protective activity of complexing type inhibitors for acidic medium has been proposed and extensively studied over the last decade [6, 7]. In such systems, protective layers are formed as a result of formation of the slightly soluble complexes of added organic ligands and the metal cations. Deposition of such compounds leads to the formation of the phase metalorganic layers on the metal surface and subsequent decrease in corrosion rate. Thus, application of complexing type inhibitors allows one to implement in situ surface modification for obtaining hybrid protective and functional coatings.

Polyhexamethylene guanidine (PHMG) showed high inhibitive efficiency for protection of carbon steel in acidic medium [6, 7, 13, 14] while exhibiting moderate performance in neutral aqueous saline solutions. Gravimetric measurements showed that PHMG provides 40% corrosion protection at the concentration of 30 mmol/l.

Relatively low efficiency of sole PHMG may be attributed to the fact that according to the protection mechanism of complexing type inhibitors, the corrosion process should provide sufficient amount of Fe^2+^ ions for the complexation. Suchlike state is spontaneously established and maintained in acidic medium, while in neutral aqueous saline solutions, such conditions should be engineered according the Pourbaix diagram for the particular system. Hence, sodium nitrite can stimulate the localized pitting attack; it may indirectly facilitate to establishing of the favorable conditions and engage the complexing type component of the inhibitive mixture. In addition, PHMG protection performance tends to increase under elevated temperature conditions that being combined with NaNO_2_ may further improve the efficiency of such mixture.

**Fig. 5:**
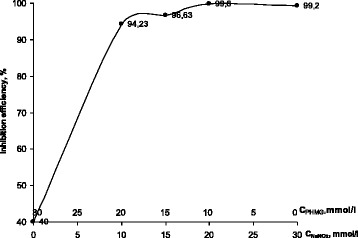
Dependence of the inhibition efficiency for carbon steel in the aqueous saline solution on the ratio of molar concentrations of the individual components in isomolar mixtures NaNO_2_-PHMG (the total concentration of the components—30 mmol/l)

The isomolar series method showed a sharp increase in the protective efficiency with addition of sodium nitrite while the peak value of 99.8% is reached at the concentration ratio NaNO_2_-PHMG as 2:1 (Fig. 5). Polarization measurements are found to be in a good agreement with gravimetrical tests. Analysis of the polarization curves showed that electrochemical behavior of the NaNO_2_-PHMG mixture is close to sole NaNO_2_ with extended passive zone in a wide range of potentials at the early stages of the measurements, while acting similar to PHMG at the later stages.

**Fig. 6:**
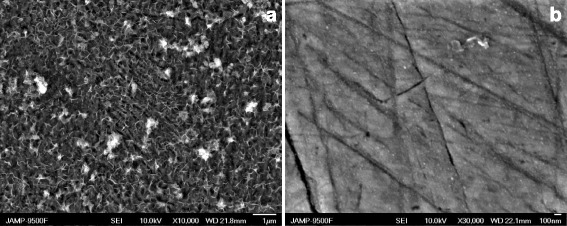
SEM images of the carbon steel surface after exposition in solutions with **a** PHMG (30 mmol/l) and **b** NaNO_2_ (20 mmol/l) and PHMG (10 mmol/l). Exposure time, 168 h

**Fig. 7:**
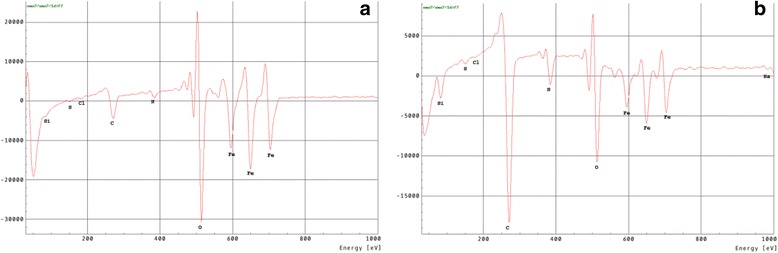
Auger spectra of the protective films in the presence of **a** PHMG (30 mmol/l) and **b** NaNO_2_ (20 mmol/l) and PHMG (10 mmol/l)

Investigation of the surface morphology after immersion in the solution with PHMG (Fig. 6a) expectedly shows significant amount of corrosion products mixed with organic inclusions that is also consistent with the Auger spectroscopy results (Fig. 7a, Table 1). Whereas in the case of NaNO_2_-PHMG mixture, the surface morphology is in almost pristine condition (Fig. 6b) while the elemental composition of the surface layer indicates presence of the protective film of mainly organic nature (Fig. 7b, Table 1).

**Table 1:** Key to Fig. 7: elemental composition of the protective films with Auger

Figure	C	N	O	Na	Si	S	Cl	Fe
7a	12.3	5.5	55.6	0.0	0.1	0.1	0.1	26.4
7b	48.1	16.1	22.3	0.5	1.8	0.3	0.1	10.9

All values are in atomic %

Further analysis of the Auger spectra indicates the formation of nanoscaled protective film as a result of tandem action of the mixture components where NaNO_2_ facilitates establishing the conditions for the complexation process and formation of metalorganic layers with PHMG-Fe complexes. According to the hybrid QM/MM calculations [14], the PGMG-Fe^2+^ complexes geometry will contribute to the formation of irregular three-dimensional mesh frameworks that will determine the sub-nanoscaled structure of the phase metalorganic layers.

## Conclusions

Regularities and mechanism of the protective layers formation in presence of synergistic mixtures of the corrosion inhibitors with different mechanism of action have been investigated. The isomolar series method has been employed for determination of optimal concentration ratios of the mixture components. For the NaNO_2_-Na_2_SiO_3_ mixture, the maximum protective efficiency of *z* = 99.97% is achieved at the concentration ratio as 1:2, while for NaNO_2_-PHMG system *z* = 99.8% at the concentration ratio as 2:1.

It was found that NaNO_2_-Na_2_SiO_3_ mixture demonstrates much improved anticorrosive behavior compared to sole sodium nitrite due to suppressing the pitting corrosion. The NaNO_2_-PHMG mixture ensures the formation of thin metalorganic layers with PHMG-Fe complexes and provides better protection under elevated temperature conditions.

The mechanism of joint action of the NaNO_2_-PHMG inhibitive mixture for protection of carbon steel in aqueous saline medium has been proposed. Sodium nitrite may indirectly facilitate to the establishing of the favorable conditions for the complexation process and engage the complexing type component of the mixture.

The inhibitive mixtures with such design where one component tuned to promote the most efficient realization of the other component protective mechanism may be distinguished to a dedicated class of tandem inhibitors.

On the basis of the proposed mechanism, the advanced surface engineering methods for obtaining nanoscaled and sub-nanostructured functional coatings may be developed.
